# Dispelling the remoteness myth- a geospatial analysis of where out-of-hospital cardiac arrests are occurring in Western Australia

**DOI:** 10.1016/j.resplu.2024.100805

**Published:** 2024-10-21

**Authors:** Ashlea Smith, Judith Finn, Karen Stewart, Stephen Ball

**Affiliations:** a Prehospital, Resuscitation and Emergency Care Research Unit (PRECRU), Curtin School of Nursing, Curtin University, Bentley, Western Australia, Australia; b St John Western Australia, Western Australia, Australia; c Department of Epidemiology and Preventive Medicine, Monash University, Victoria, Australia; d Emergency Medicine, Medical School, The University of Western Australia, Perth, Australia

**Keywords:** Emergency medical services, Rural health, Epidemiology, Emergency medicine, Spatial analysis

## Abstract

**Background:**

Rurality has been shown to have a strong effect on survival from out-of-hospital cardiac arrest (OHCA), with survival in rural areas approximately half that of metropolitan areas. Western Australia provides a unique landscape to understand the impact of rurality, with 2.6 million people spread across 2.5 million km^2^. We conducted a scale geospatial analysis with respect to population density and proximity to services, to understand the impact of rurality on bystander interventions, prehospital management and survival of OHCA patients.

**Methods:**

We conducted a retrospective cohort study with a geospatial analysis of ambulance-attended, medical OHCA cases from 2015 to 2022. We compared bystander interventions, distances to services, population density and survival outcomes, stratified by a four-scale regional (broad scale) categorisation of rurality, and proximity to town scale.

**Results:**

There were a total of 6,763 cases within the study cohort (Major Cities- 5,186, Inner Regional- 605, Outer Regional-599 and Remote- 373). The majority of OHCAs occurred within towns, and within close proximity to people and health services. Bystander interventions were higher for more remote cases. Increased distance from town was associated with a 5 % decrease per kilometre in the odds of Return of Spontaneous Circulation (ROSC) on arrival at hospital (OR = 0.95 [95 % Confidence Interval 0.92–0.98]). Despite close proximity to ambulance services, ambulance response times were more prolonged with increasing remoteness.

**Conclusions:**

OHCA cases within regions classified as Regional and Remote typically occurred within towns, and in close proximity to emergency services. However, ambulance response times within rural and remote towns were long relative to their proximity to ambulance stations. These findings provide a new perspective on the issue of remoteness for OHCA cases.

## Introduction

Rurality presents a clear challenge for survival from out-of-hospital cardiac arrest (OHCA), with a 2023 systematic review showing that internationally, the odds of OHCA survival in rural areas are approximately half that of metropolitan areas.^1^ While some factors, such as access to tertiary hospitals, are likely to have broad-scale/regional effects on OHCA survival, there may be other factors earlier in the Chain of Survival,^2^ such as ambulance response time and bystander interventions (e.g., cardiopulmonary resuscitation [CPR], and defibrillation from an automated external defibrillator [AED]) that operate at a much finer spatial scale. Thus, an understanding of the impacts of rurality on OHCA may benefit from examining the issue of rurality at multiple spatial scales.

Using the geographical context of Western Australia, with a vast land space of 2.5 million km^2^ and only one major city,^3^ we hypothesised that beyond a spatially broad, regional classification of rurality (as metropolitan, inner regional, outer regional, and remote areas), differences in OHCA patient care and survival may additionally be evident at a more granular scale, in relation to the proximity to the nearest township. Therefore, in this paper we aimed to understand the geographical context of OHCA cases at multiple spatial scales, and to examine the implications of geographic context for bystander interventions, ambulance travel times and OHCA patient survival.

## Methods

### Study design

We conducted a geospatial analysis of ambulance-attended, medical^4^ OHCA cases between 1 January 2015 and 31 December 2022 in Western Australia (WA), using a retrospective cohort design. Emergency medical service (EMS)-witnessed cases were excluded from this cohort. Analyses were stratified according to a four-category rurality scale^5^ (see “Rurality Definition” below) and further stratified by proximity to the boundary of the nearest town. Based on this two-level stratification, we compared patient characteristics, bystander CPR, bystander delivery of an automated external defibrillator (AED) shock, distance to the nearest ambulance station, ambulance response time (call answer to ambulance arrival on scene), distance and travel time to the initial destination hospital, the proportion of cases with return of spontaneous circulation (ROSC) on arrival at the emergency department (ED), and the proportion surviving to 30 days.

Ethical approval for this study was granted from the Curtin Human Research Ethics Committee (Approval no. HR128/2013), and by the St John WA Research Governance Committee. This study was reported in accordance with the STROBE guidelines.^6^

### Study setting

A total of 2.6 million people occupied WA’s 2.5 million square kilometres in 2020.^7^ Approximately 77 % of the population lives within the capital city, Perth,^8^ which spans 6400 km^2^,^7^ with an average population density of 300 people per km^2^. Outside Perth are 500,000 people (0.2 people per km^2^) who primarily reside in regional towns.^7^.

St John WA (SJ-WA) provides ambulance services for nearly all of WA, except for an area in the north-west of WA that represents 0.4 % of the total population (not included in this study). Within metropolitan Perth, ambulances are staffed by registered paramedics with advanced life support skills capability. Clinical Support Paramedics and Critical Care Paramedics with intensive care level training are also available to assist paramedics in high acuity cases in metropolitan Perth (including OHCA cases), carrying a LUCAS device for mechanical compressions.^9^ In rural WA, each crew has either a volunteer Emergency Medical Technician (EMT) and paid paramedic, or two volunteer EMTs (Appendix 1), with volunteer EMTs having basic life support (BLS) capability. Rural crews are also supported by other clinical resources, such as Community Paramedics. The crews are dispatched from their station (Fig. 2), or the closest location; volunteer EMT crews operate on an “on-call” basis, and may respond from within the community.

The hospital system in WA is composed of a variety of services, from tertiary hospitals to community nursing posts.^10^ Currently, the only tertiary hospitals and cardiac catheterisation units (CCU) with 24-hour service in WA are Perth.^11^ In rural WA, there are no public CCUs, and some smaller hospitals and nursing posts do not provide a 24 h service.^11^ Larger rural hospitals provide telehealth and visiting specialist services.

### Data sources

The cohort data were sourced from the SJ-WA OHCA Database. This is managed by the Prehospital, Resuscitation and Emergency Care Research Unit (PRECRU) at Curtin University, WA, and includes all SJ-WA ambulance-attended OHCA cases in WA. The data are comprised of a combination of electronic patient care records (ePCR) as entered by paramedics/ambulance officers, and computer-aided dispatch (CAD) data. The database variables are collected in line with the international Utstein definitions,^4^ and include patient age and sex, witness status (bystander-witnessed, EMS-witnessed, unwitnessed), bystander interventions (bystander CPR [B-CPR], bystander AED shock delivered), initial arrest rhythm, ambulance response time, and geographic coordinates of dispatch location (latitude and longitude, using the Geocentric Datum of Australia 94 [GDA94]). The primary outcome of interest in this study is return of spontaneous circulation (ROSC) on arrival at the Emergency Department (ED); with a secondary outcome of 30-day survival (ascertained from the WA Death Registry^12^). SJ-WA ambulance station locations and hospital locations were provided by SJ-WA, in GDA94 coordinates.

### Rurality definition

We defined rurality in this study using the Australian Standard Geographic Classification- Remoteness Areas (ASGC-RA).^13^ The ASGC-RA is a classification of remoteness developed from the Accessibility/Remoteness Index of Australia (ARIA + ), a continuous measure of remoteness derived from indexing of road distance to the closest available services (e.g., health care and retail).^5^ This scale has five categories: Major Cities, Inner Regional, Outer Regional, Remote and Very Remote.^5^ Due to low case numbers in the Remote and Very Remote categories in this study, we combined these as Remote.

To assess proximity to towns, we used the Urban Centres and Localities (UCL) geography (obtained from the Australian Bureau of Statistics^14^) to define town boundaries. We refer to UCLs as ‘towns’ throughout this study.

### Statistical analysis

Spatial analyses were undertaken using ArcGIS Desktop,^15^ with coordinates projected on the Map Grid of Australia 1994. The remoteness category for each OHCA case (as per the ASGC-RA classification described above) was determined using the Spatial Join function in ArcGIS Spatial Analyst, (v. 10.8.2, Esri). We measured the proximity of each OHCA case to the boundary of the nearest town, and used this to generate three proximity categories: within a town, within 10 km of a town boundary and greater than 10 km of a town boundary. Road centreline data were extracted from OpenStreetMap,^16^ and road distances to services calculated using ArcGIS Network Analyst.^17^ The minimum distance by road from each OHCA case to: 1) the nearest ambulance station, 2) nearest hospital and 3) nearest tertiary hospital were measured. Using a Spatial Join in ArcGIS Spatial Analyst, we ascertained the average population density in the census area surrounding each individual OHCA case, using Australian Bureau of Statistics’ “SA1” geography,^18^ which is the smallest geographical unit used for census data in Australia.^19^.

Using STATA,^20^ we compared (across rurality categories and town proximity categories) median road distance from arrest location to the nearest ambulance station, and the median road distance from arrest location to initial destination hospital, as well as patient and arrest characteristics, population density, and rates of ROSC at ED and 30-day survival. Comparisons were made using Chi-squared tests for categorical variables, and ANOVA for continuous variables. P-values less than 0.05 were considered statistically significant.

Variables were entered simultaneously^21^ into multivariable logistic regression models to ascertain the effect of rurality and proximity to town on the odds of ROSC at ED and 30-day survival. Three models were produced, namely (1) an unadjusted model, (2) a model adjusted for non-modifiable factors − i.e., age, sex, initial arrest rhythm (shockable/non-shockable), type of location of arrest (public/private residence/residential aged care/other) and witness status (unwitnessed/bystander witnessed), and (3) a model adjusted for non-modifiable factors, as well as B-CPR and AED use. Response time was not included in these models, as we were specifically interested in measuring the effect of proximity to town as a main variable of interest. With response time being a likely major component of any effect of proximity on survival (i.e. as mediator), its inclusion in the models would absorb some of our effect of interest.

## Results

**Fig. 1:**
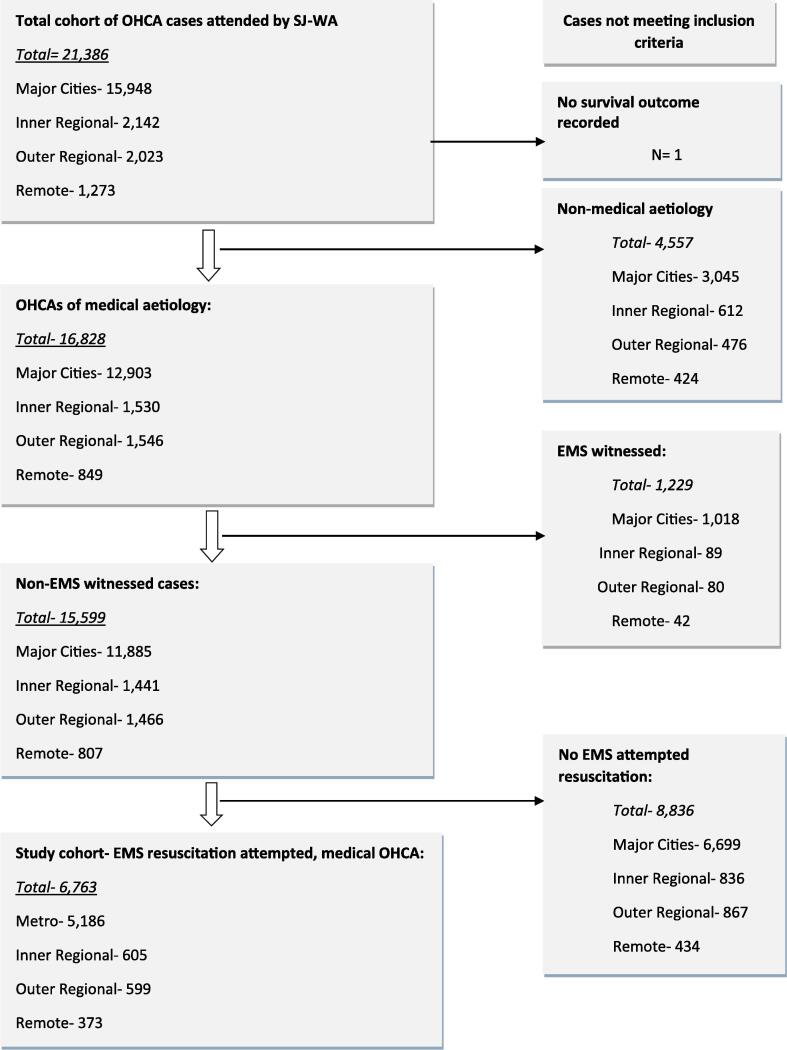
Study cohort selection flow chart.

Between 1 January 2015 and 31 December 2022, there was a total of 15,599 EMS-attended medical OHCA cases in Western Australia (Major Cities 11,885; Inner Regional 1,441; Outer Regional 1,466; Remote 807) (Fig. 1). Of these, 6,763 cases (43.3 %) comprised our study cohort − i.e. cases where resuscitation was attempted by the EMS. The proportion of OHCAs with EMS resuscitation attempts ranged from 40.2 % to 48.6 % across all rurality categories, and categories of proximity to town (Table 1). The study cohort of 6,763 resuscitation-attempted cases comprised 5,186 cases in Major Cities, 605 cases in Inner Regional, 599 cases in Outer Regional and 373 cases in Remote areas.

**Fig. 2:**
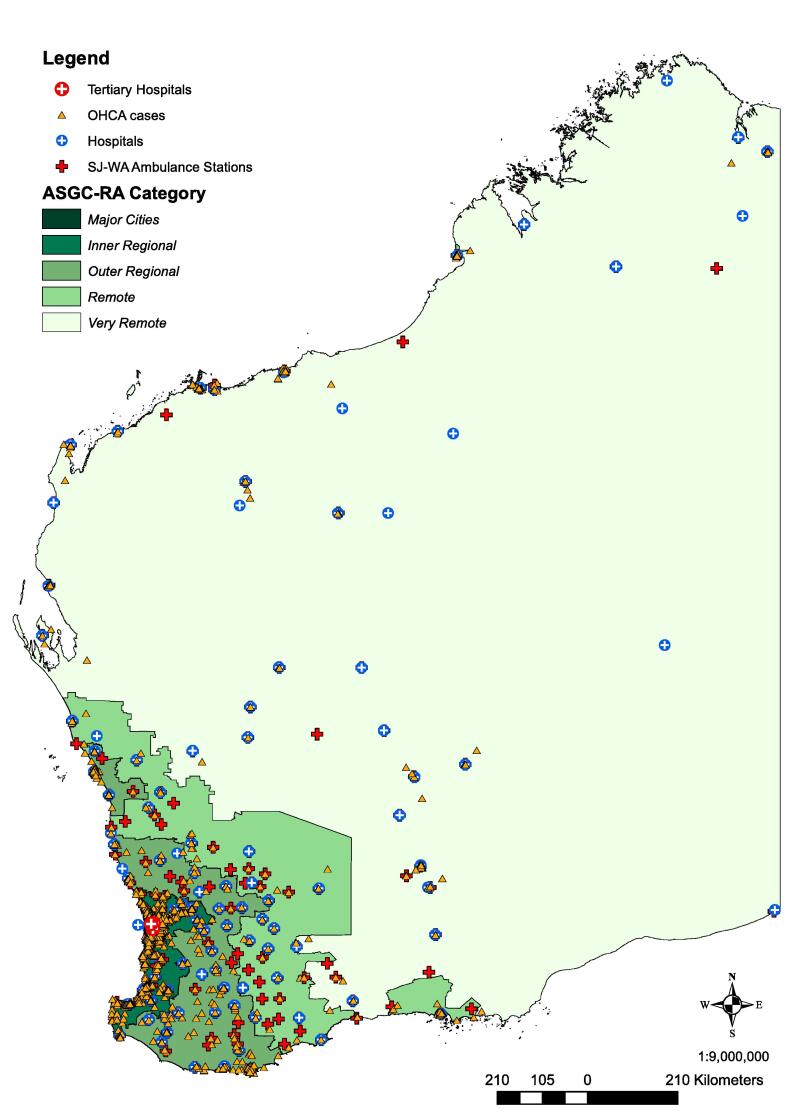
Location of medical out-of-hospital cardiac arrest cases with resuscitation attempted by St John WA in Western Australia.

**Table 1:** Characteristics of EMS attended OHCA cases stratified by rurality category and proximity to town.

	**Major cities**				**Inner Regional**				**Outer Regional**				**Remote**			
**Town Proximity**	**Within town**	**Within 10 km**	**10 km+**	**P Value**	**Within town**	**Within 10 km**	**10 km+**	**P Value**	**Within town**	**Within 10 km**	**10 km+**	**P value**	**Within town**	**Within 10 km**	**10 km+**	**P value**
**All medical cases attended by EMS**																
**Total cases**	11,700	185	NA		1,104	276	61		1,162	158	146		616	64	127	
**EMS resuscitation attempted,** **n (%)**	5,096 (43.5)	90 (48.6)	NA	0.166	464 (42.0)	111 (40.2)	30 (49.2)	0.438	471 (40.5)	69 (43.6)	59 (40.4)	0.748	292 (47.4)	29 (45.3)	52 (40.9)	0.409
**EMS resuscitated cases of medical aetiology**																
**Total cases, n (%)**	5,096 (98.2)	90 (1.8)	NA		464 (76.6)	111 (18.3)	30 (4.9)		471 (78.6)	69 (11.5)	59 (9.8)		292 (78.2)	29 (7.7)	52 (13.9)	
**Average population density (persons per km^2^)**	2106.78	38.02	NA	<0.001	871.20	6.08	3.04	<0.001	645.93	1.74	0.35	<0.001	633.53	0.14	0.07	<0.001
**Age**	68 (54, 79)	65 (55, 77)	NA	0.016	68 (54, 78)	63 (52, 72)	67.5 (51, 76)	<0.001	66 (52, 78)	64 (54, 75)	63.5 (55, 73)	0.005	57 (44, 68)	69 (59, 76)	61 (52, 70)	<0.001
**Sex (male),** **n (%)**	3,446 (67.6)	73 (81.1)	NA	0.007	325 (70.0)	79 (71.1)	22 (73.3)	0.912	310 (65.8)	49 (71.0)	45 (76.2)	0.216	189 (64.7)	23 (79.3)	41 (78.8)	0.051
**Witness status, n (%)**																
*Unwitnessed*	2,436 (47.8)	41 (45.5)	NA	0.672	230 (49.5)	47 (42.3)	11 (36.6)	0.184	248 (52.6)	31 (44.9)	24 (40.7)	0.108	150 (51.3)	16 (55.1)	18 (34.6)	0.068
*Bystander witnessed*	2,660 (52.2)	49 (54.4)	NA		234 (50.4)	64 (57.6)	19 (63.3)		223 (47.3)	38 (55.1)	35 (59.3)		142 (48.6)	13 (44.8)	34 (65.4)	
**Bystander CPR, n(%)**																
*Total*	3,685 (72.3)	79 (87.8)	NA	0.001	364 (78.4)	96 (86.5)	24 (80.0)	0.164	349 (74.1)	59 (85.5)	51 (86.4)	0.019	218 (74.6)	24 (82.7)	48 (92.3)	0.015
*Bystander witnessed*	2,028 (76.2)	44 (76.5)	NA	0.027	192 (82.1)	58 (90.6)	16 (84.2)	0.254	174 (78.0)	35 (92.1)	30 (85.7)	0.092	111 (78.2)	13 (100)	32 (94.1)	0.020
**Bystander AED, n(%)**																
*Total*	183 (3.6)	1 (1.1)	NA	0.207	24 (5.2)	8 (7.2)	3 (10.0)	0.425	13 (2.7)	4 (5.8)	4 (6.8)	0.156	13 (4.4)	5 (17.2)	5 (9.6)	0.013
*Bystander witnessed*	164 (6.2)	0 (0)	NA	0.073	18 (7.7)	6 (9.4)	3 (15.8)	0.460	8 (3.6)	3 (7.9)	2 (5.7)	0.449	8 (5.6)	5 (38.4)	5 (14.7)	<0.001
**Died at scene, n(%)**	2,393 (46.9)	44 (48.9)	NA	0.928	226 (48.7)	66 (59.4)	21 (70.0)	0.015	223 (47.3)	38 (55.0)	42 (71.2)	0.002	103 (35.2)	14 (48.3)	32 (61.5)	0.001
**Distance to EMS station, km, med (IQR)**	4.0 (2.5, 5.8)	8.6 (6.4, 11.5)	NA	<0.001	3.0 (1.7, 5.6)	10.6 (5.8, 14.8)	23.5 (18.8, 25.9)	<0.001	2.6 (1.3, 4.4)	9.1 (5.3, 12.0)	25.8 (16.2, 31.0)	<0.001	2.1 (0.9, 3.9)	5.7 (3.0, 8.1)	25.1 (14.3, 36.5)	<0.001
**EMS response time, mins, med (IQR)**	8.9 (6.9, 11.4)	12.1 (10.0, 15.8)	NA	0.799	10.7 (7.9, 15.5)	19.3 (13.7, 24.0)	24.4 (20.8, 33.3)	<0.001	11.2 (8.0, 15.9)	18.9 (13.6, 24.3)	31.7 (26.0, 39.7)	0.011	12.2 (8.7, 16.6)	16.9 (13.3, 20.2)	36.9 (26.0, 49.3)	0.090
**Distance to nearest hospital, km, med (IQR)**	8.5 (5.3, 11.2)	12.5 (9.7, 16.1)	NA	<0.001	4.6 (2.5, 14.1)	23.3 (15.7, 31.5)	38.1 (23.5, 40.1)	<0.001	2.8 (1.2, 5.5)	10.9 (7.3, 14.7)	31.6 (25.4, 39.2)	<0.001	1.8 (1.1, 3.1)	7.2 (5.2, 11.9)	37.0 (23.3, 54.2)	<0.001
**Distance to actual hospital, km, med (IQR)**	8.6 (5.3, 12.2)	14.0 (9.4, 19.7)	NA	0.003	4.4 (2.3, 10.8)	23.1 (14.8, 32.2)	36.1 (19.8, 39.8)	<0.001	3.0 (1.2, 5.4)	9.7 (3.2, 13.5)	29.8 (22.5, 35.9)	0.001	1.9 (1.2, 3.3)	6.2 (5.6, 11.9)	36.7 (22.8, 50.6)	0.004
**Distance to tertiary hospital, km, med (IQR)**	12.8 (8.1, 22.5)	28.7 (22.5, 41.4)	NA	<0.001	153.0 (137.4, 183.2)	82.6 (49.1, 169.4)	96.7 (88.8, 172.5)	<0.001	404.7 (276.6, 416.9)	339.2 (215.3, 401.3)	248.6 (156.2, 356.4)	0.007	1512.0 (667.8, 1598.8)	663.5 (560.6, 1234.3)	457.1 (319.0, 829.6)	0.652
**EMS transport to hospital time, mins, med (IQR)**	10.0 (6.9, 13.2)	11.7 (9.4, 17.1)	NA	0.011	6.9 (4.3, 11.0)	19.5 (12.9, 31.8)	23.0 (15.0, 24.8)	0.150	5.2 (3.0, 8.6)	11.0 (5.0, 14.0)	20.7 (16.0, 34.0)	0.212	4.9 (2.5, 7.0)	7.0 (4.0, 10.9)	25.4 (13.6, 31.7)	<0.001
**ROSC at ED, n (%)**	953 (18.7)	16 (17.8)	NA	0.824	60 (12.9)	14 (12.6)	1 (3.3)	0.302	52 (11.0)	6 (8.7)	3 (5.1)	0.329	31 (10.6)	0 (0)	4 (7.7)	0.157
**Survival to 30 days, n(%)**	447 (8.7)	10 (11.1)	NA	0.438	37 (7.9)	8 (7.2)	0 (0)	0.271	26 (5.5)	2 (2.9)	3 (5.1)	0.656	17 (5.8)	0 (0)	4 (7.7)	0.338

### Geographical context

**Fig. 3:**
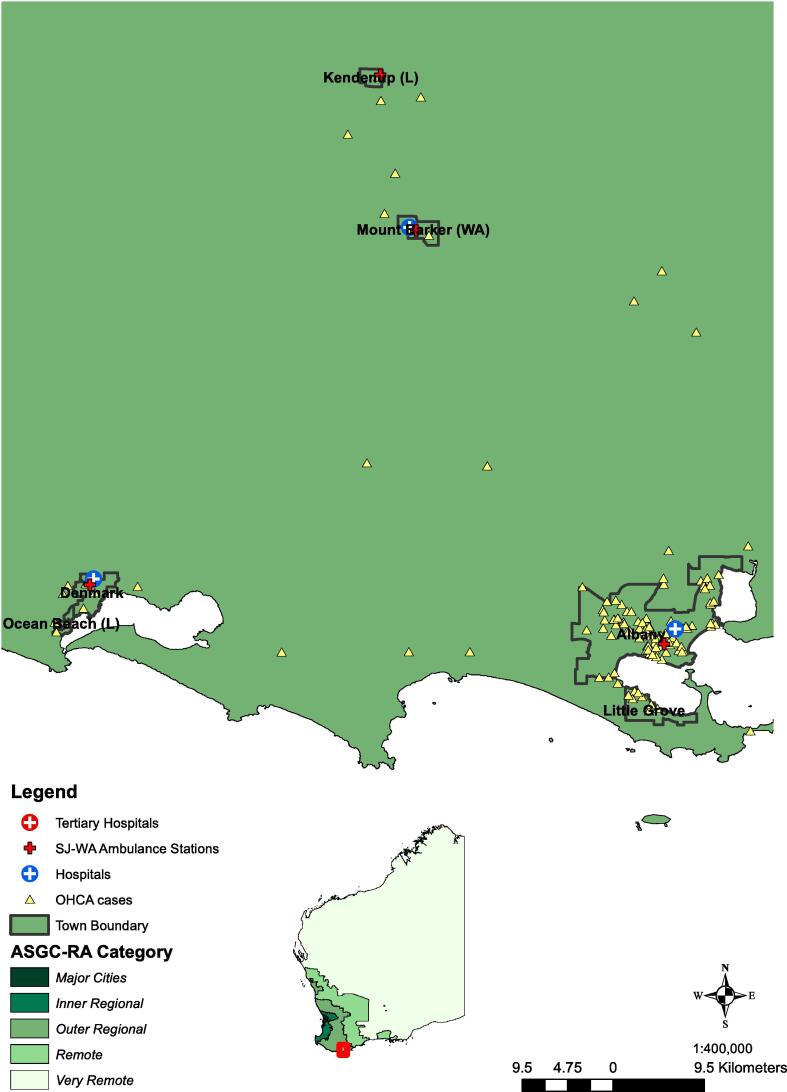
Example of obfuscated location of rural OHCA cases relative to town boundaries.

Fig. 2 depicts the spread of cases across WA, and their corresponding rurality categories, with Fig. 3 demonstrating the location of cases relative to town boundaries in part of rural WA (case locations were geomasked for display purposes, using random perturbation of up to 250 m).^22^

**Fig. 4:**
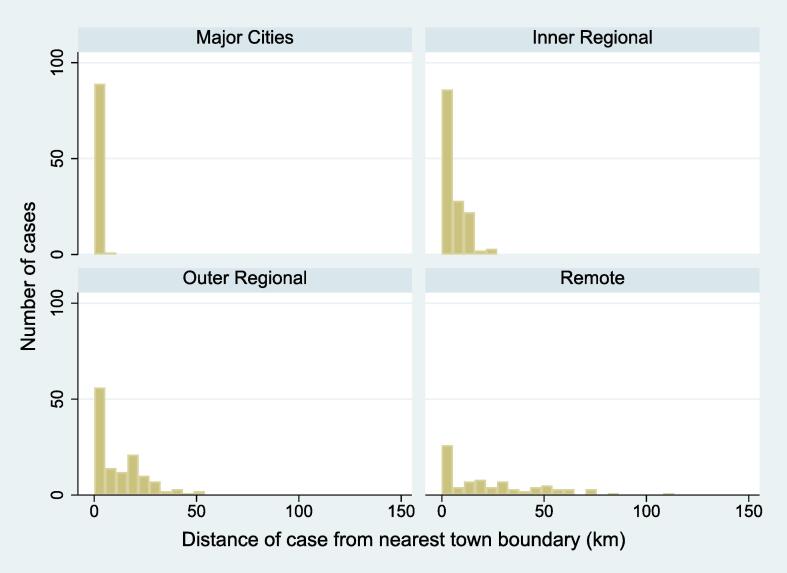
Distance of OHCA cases from the nearest town boundary according to rurality category.

The majority of cases, irrespective of rurality category, occurred within a town (Major Cities 98.2 %; Inner Regional 76.6 %; Outer Regional 78.6 %, and Remote 78.2 %). Remote areas had the largest variation in distance to town, with an interquartile range of 3.4 km to 43.7 km (median 18.1 km). Fig. 4 shows, for each rurality category, histograms of the distance to the nearest town for those cases outside of a town. Within towns, the average population density around cases was highest in Major Cities (2106.78 people per km^2^), decreasing with increasing remoteness (Inner Regional 871.20; Outer Regional 645.93; Remote 633.53, p=<0.001). The lowest average surrounding population density occurred for cases that were more than 10 km from a town boundary in Remote areas (0.07 people per km^2^).

For cases occurring within a town, the median distance to both ambulance stations (Major Cities 4.0 km, Inner Regional 3.0 km, Outer Regional 2.6 km, Remote 2.1 km, p=<0.001) and the nearest ED (Major Cities 8.6 km, Inner Regional 4.4 km, Outer Regional 3.0 km, Remote 1.9 km, p=<0.001) decreased with increasing remoteness. In contrast, for cases occurring outside of a town, the median distance from cases to ambulance stations and the nearest ED increased with increasing remoteness (Table 1).

### Bystander and ambulance response

For all remoteness categories, the lowest rate of B-CPR occurred within towns (Major Cities 72.3 %, Inner Regional 78.4 %, Outer Regional 74.1 %, Remote 74.6 %), and the highest rate was observed in cases greater than 10 km from town in Remote areas (92.3 %, p = 0.015). B-CPR rates were consistently higher in all remoteness categories when compared to Major Cities for bystander-witnessed cases, with the highest rate observed in Remote areas within 10 km from a town boundary (100 %, p = 0.020). Bystander AED use increased with increasing remoteness and distance from town (Table 1). The highest rates of bystander AED use were in Inner Regional areas greater than 10 km of a town boundary (10.0 %), and in Remote areas, both within 10 km of a town boundary (17.2 %) and greater than 10 km from a town boundary (9.6 %). For bystander-witnessed cases, the highest observed rates of AED use were within both 10 km of, and greater than 10 km from towns in Remote areas (38.4 % and 14.7 % respectively, p=<0.001).

The median ambulance response time was lowest within towns across the remoteness categories (Major Cities 8.9 mins, Inner Regional 10.7, Outer Regional 11.2, Remote 12.2). With increasing distance from town, response time was progressively longer across all remoteness categories.

### Impact on survival

The proportion of cases that achieved ROSC at ED was highest in Major Cities (18.7 %), with a declining proportion with increasing remoteness and distance to town (Table 1). The lowest rates of ROSC at ED were in cases greater than 10 km from town (Inner Regional 3.3 %, Outer Regional 5.1 %, Remote 7.7 %).

Thirty-day survival was highest both within town and within 10 km of town in Major Cities (8.7 % and 11.1 %, respectively). The lowest rates of 30-day survival occurred in cases greater than 10 km from town, with no recorded 30-day survival in Inner Regional areas, 5.1 % in Outer Regional and 7.7 % in Remote areas.

### Multivariable logistic regression modelling

#### ROSC at ED

After adjusting for non-modifiable factors, the odds of ROSC at ED reduced with increasing rurality when compared to Major Cities (Inner Regional OR = 0.57 [95 %CI 0.43–0.75], Outer Regional OR = 0.51 [95 %CI 0.38–0.68], Remote OR = 0.41 [95 %CI 0.28–0.60]) (Table 2). After additionally adjusting for modifiable factors, the odds of ROSC at ED did not increase (Inner Regional OR = 0.60 [95 %CI 0.47–0.76], Outer Regional OR = 0.50 [95 %CI 0.38–0.65], Remote OR = 0.42 [95 %CI 0.30–0.58]). After full adjustment, the odds of ROSC at ED decreased by 5 % for every additional kilometre from town (OR = 0.95 [95 %CI 0.92–0.98]).

**Table 2:** Multivariable analysis of EMS resuscitation attempted, medical OHCA cases.

	**ROSC at ED- OR (95 %CI)**			**30-day Survival- OR (95 %CI)**		
Variable	**Unadjusted**	**Model 1**^i^	**Model 2**^ii^	**Unadjusted**	**Model 1**^i^	**Model 2**^ii^
**Rurality category**						
Major Cities	Ref.			Ref.		
Inner Regional	0.61 (0.47–0.79)	0.57 (0.43–0.75)	0.60 (0.47–0.76)	0.83 (0.60–1.14)	0.77 (0.53–1.10)	0.75 (0.52–1.08)
Outer Regional	0.49 (0.37–0.64)	0.51 (0.38–0.68)	0.50 (0.38–0.65)	0.56 (0.39–0.82)	0.62 (0.41–0.94)	0.62 (0.41–0.94)
Remote	0.45 (0.31–0.64)	0.41 (0.28–0.60)	0.42 (0.30–0.58)	0.61 (0.39–0.97)	0.51 (0.31–0.86)	0.52 (0.31–0.87)
**Proximity to town (effect per additional km)**	0.95 (0.92–0.98)	0.96 (0.94–0.99)	0.95 (0.92–0.98)	0.98 (0.95–1.00)	0.98 (0.95–1.01)	0.97 (0.94–1.00)
**Age (effect per additional year of age)**	0.99 (0.98–0.99)	0.98 (0.98–0.99)	0.98 (0.98–0.99)	0.98 (0.97–0.98)	0.97 (0.96–0.97)	0.97 (0.96–0.97)
**Sex**						
Female	Ref.			Ref.		
Male	1.05 (0.91–1.20)	0.73 (0.63–0.85)	0.79 (0.69–0.90)	1.89 (1.53–2.34)	1.11 (0.87–1.42)	1.12 (0.87–1.43)
**Location**						
Private residence	Ref.			Ref.		
Residential care facility	0.58 (0.42–0.82)	0.82 (0.57–1.16)	0.80 (0.58–1.11)	0.15 (0.05–0.40)	0.43 (0.15–0.99)	0.43 (0.15–1.19)
Public	2.67 (2.28–3.12)	1.58 (1.32–1.89)	1.16 (0.99–1.35)	5.04 (4.17–6.09)	2.23 (1.79–2.76)	2.14 (1.69–2.70)
Otheriii	1.64 (1.18–2.28)	1.48 (1.04–2.13)	1.30 (0.93–1.81)	1.81 (1.15–2.86)	1.31 (0.79–2.17)	1.27 (0.75–2.14)
**Witness Status**						
Unwitnessed	Ref.			Ref.		
Bystander	3.67 (3.17–4.25)	2.54 (2.17–2.98)	2.09 (1.82–2.39)	5.82 (4.59–7.39)	2.74 (2.10–3.57)	2.69 (2.07–3.52)
**Initial rhythm**						
Non-shockable	Ref.			Ref.		
Shockable	5.29 (4.63–6.05)	3.75 (3.23–4.36)	3.36 (2.92–3.87)	19.56 (15.51–24.68)	12.28 (9.54–15.80)	11.78 (9.11–15.23)
**Bystander CPR**						
No	Ref.			Ref.		
Yes	1.73 (1.47–2.03)		1.09 (0.97–1.72)	3.45 (2.59–4.58)		1.56 (1.14–2.14)
**Bystander AED**						
No	Ref.			Ref.		
Yes	4.21 (3.27–5.43)		1.29 (0.98–1.72)	6.89 (5.26–9.04)		0.97 (0.70–1.34)

i Model 1- Adjusted for rurality category, proximity to town and non-modifiable factors (age, sex, location, witness status, initial rhythm).

ii Model 2- Adjusted for rurality category, proximity to town, non-modifiable factors (age, sex, location, witness status, initial rhythm) and modifiable factors (bystander CPR and bystander AED use).

iii Other location- e.g., prisons, medical centres.

#### 30-day survival

When adjusted for non-modifiable factors, the odds were reduced for Outer Regional (OR = 0.62 [95 %CI 0.41–0.94]) and Remote areas (OR = 0.51 [95 %CI 0.31–0.86]). When fully adjusted, the odds of 30-day survival were largely unchanged. There was no statistically significant effect of proximity to town on 30-day survival, after full adjustment.

## Discussion

Contrary to our expectation that OHCAs occurring in rural areas would occur far from resources and would be in areas of low population density, a high proportion of the rural arrests (and metro) in our cohort occurred within a town, and within close proximity to people, ambulance services, and hospitals. Despite this, ambulance response times were still prolonged in rural areas compared to metro. The percentage of EMS resuscitation attempts was relatively consistent across remoteness categories and in relation to distance to town. We observed a higher proportion of bystander interventions in rural areas, particularly in areas classified as Remote. However, remoteness was still associated with reduced odds of ROSC at ED and 30-day survival, even after adjusting for known prognostic factors. Importantly, proximity to town was a prominent factor for ROSC, with increasing distance to town associated with a significant decline in the odds of ROSC at ED. These results redefine our understanding of the geographical context of OHCA in relation to remoteness, demonstrating that OHCAs are not necessarily as far away from resources as spatially-broad categories of remoteness might suggest.

In addition to our finding that the majority of rural OHCAs were within towns, we found that among the OHCAs in Western Australian that occurred within towns, rural cases had much shorter median distances to ambulance stations and hospitals, compared to OHCA cases in Major Cities. In combination, these results mean that a large proportion of rural OHCA cases in Western Australia, while occurring in regions classified as regional or remote at a broad scale, are typically very close to emergency services. These results demonstrate the value of analysing the rurality of OHCA at multiple spatial scales, and help re-define our understanding of what rurality means for out-of-hospital cardiac arrest.

While most rural cases occurred within close proximity to services, there was a significant impact on patient survival for those cases with increasing distances from towns. We observed a 5 % reduction in the odds of ROSC at ED with every additional kilometre from a town to an OHCA scene. Reduced ambulance travel distances have been previously found to be associated with improved OHCA survival.^23^, ^24^ While Hansen et al.^25^ found that closer proximity of OHCAs to first responders resulted in improved survival to hospital discharge, even after adjusting for B-CPR and AED use within our study, there was no significant improvement in the odds of 30-day survival. However, the lack of a significant improvement in 30-day survival may be a result of a type 2 error, given the low sample size. Rural OHCA patients in our study were also required to travel extensive distances to receive tertiary care. Tranberg et al.^26^ advocate for the prioritisation of transport directly to a Cardiac Arrest Centre (CAC) for improved odds of survival. Clark et al.^27^ suggest that remotely accessed healthcare initiatives and innovative clinical approaches would allow greater access to cardiac care for those populations with limited access; however, interventional cardiology is still unavailable to these patients. Without the ability to provide equitable access to specialist facilities in rural areas, survival of OHCA may continue to be impacted.

A compelling finding from our study was the prolonged median ambulance response times relative to location of the OHCA within towns in rural areas. Delays in ambulance response have been well documented to adversely impact survival of OHCA.^28^, ^29^, ^30^ For OHCAs occurring within towns in our study, increased rurality was associated with longer response times compared to Major Cities. This is despite OHCAs in rural towns being closer to ambulance stations. Regional differences in ambulance mobilisation could provide a possible explanation for these disproportionately long response times for rural cases in our cohort. In a study of the relationship between ambulance response time and distance travelled to the scene, Lyon et al.^24^ found a time delay of five minutes at zero kilometres travelled, and hypothesised that this is due to a delay in mobilisation of ambulance resources. This phenomenon may also be a factor within our cohort, due to the requirement to mobilise volunteer crews in rural areas to respond to community emergencies. The delay may also be due to a reduced ambulance coverage within rural areas, requiring crews to travel from neighbouring towns and areas to attend the OHCA, further prolonging response time. Further research is required to understand the complexity of response time delays, given the mismatch between shorter distances and prolonged ambulance response time in rural areas.

### Limitations

The findings of this study are in the context of the Western Australian geography, and may not reflect the characteristics or rural locations or other EMS systems. We acknowledge that the skill set of the responding crews may be one of the mechanisms through which rurality has an effect on OHCA survival in Western Australia, given the increased proportion of BLS crews in rural areas. Future research could examine the relative importance of crew mix and other factors (e.g. hospital capability) as components of the overall effect of rurality on OHCA survival. We have attempted to provide granularity in our use of population density as well as stratification in location and remoteness category, however, acknowledge the complexity in defining rurality. A decision was made a priori to distinguish between OHCAs that occurred within a township, on the outskirts of a township, or distant from townships. The threshold distance of 10 km from the nearest township boundary was chosen for the latter, because it reflects an additional response time of at least 5–6 min, and therefore of large magnitude relative to the median rural response time in Western Australia of 15 min.^31^ However, we acknowledge that the choice of a threshold distance is somewhat arbitrary. The size and capability of receiving hospitals, particularly within rural areas with reduced service capability, and the in-hospital treatment given to the OHCA patients within our cohort was outside of the scope of this study, which may have an undetermined impact on 30-day survival.

## Conclusion

This study provides a finer scale geospatial analysis of the impact of rurality on OHCA than previous research. The majority of OHCAs in rural areas occurred within towns and close to services, and received a higher proportion of bystander interventions than in metropolitan areas. However, there was a significant impact of increasing distance on the odds of ROSC at ED, with lower odds of survival with increasing rurality and distance from towns. Ambulance response times were prolonged in rural areas, even within towns. Further research is required to understand the relationship between distance and response time within rural areas.

## Ethics

Ethical approval was granted from the Curtin Human Research Ethics Committee (Approval no. HR128/2013), as a sub-study to the Western Australia Prehospital Care Record Linkage Project. Approval was also granted by the St John WA Research Governance Committee.

## CRediT authorship contribution statement

**Ashlea Smith:** Writing – original draft, Visualization, Validation, Project administration, Methodology, Investigation, Formal analysis, Data curation, Conceptualization. **Judith Finn:** Writing – review & editing, Validation, Supervision, Project administration, Methodology, Investigation, Funding acquisition. **Karen Stewart:** Writing – review & editing, Supervision, Data curation. **Stephen Ball:** Writing – review & editing, Supervision, Software, Methodology, Formal analysis, Data curation, Conceptualization.

## Funding

AS is funded by Curtin University PhD Scholarship; JF is funded by NHMRC Investigator grant GTN1174838.

## Declaration of competing interest

The authors declare the following financial interests/personal relationships which may be considered as potential competing interests: AS and KS are employees of SJ-WA; SB and JF hold adjunct research positions with SJ-WA and JF receives research funding from SJ-WA. JF is on the Resuscitation Editorial Board.
