# Supplementary material for: Dispelling the remoteness myth- a geospatial analysis of where out-of-hospital cardiac arrests are occurring in Western Australia
Source: Resusc Plus. 2024 Oct 21;20:100805. doi: 10.1016/j.resplu.2024.100805 (PMC11536345; doi:10.1016/j.resplu.2024.100805)
Supplement: Supplementary Data 1 [file mmc1.docx]

Appendix 1- Ambulance crew composition by country location within cohort study time frame

| Paramedic only | Mixed crew | Volunteer only | | |
| --- | --- | --- | --- | --- |
| Bunbury | Albany  Australind  Broome  Busselton  Collie  Dawesville  East Bunbury  Geraldton  Hedland  Karratha  Kalgoorlie  Kununurra  Northam  Norseman  Pinjarra | Augusta  Balladonia  Beacon  Bencubbin  Beverley  Bindoon  Boyanup  Boddington  Bolgart  Borden  Boyup Brook  Bremer Bay  Bridgetown  Bruce Rock  Brookton  Brunswick  Bullsbrook  Calingiri  Capel  Cranbrook  Coral Bay  Cocklebiddy  Cervantes  Corrigin  Coolgardie  Chittering Gingin  Christmas Island  Chapman Valley  Carnamah  Condingup  Coorow  Cue  Cunderdin  Carnarvon  Dalwallinu  Dandaragan  Darkan  Donnybrook  Denmark  Irwin Districts  Dowerin  Dumbleyung  Dunsborough  Dwellingup  Eneabba  Esperance  Eucla  Exmouth  Frankland  Gingin | Gnowangerup  Goomalling  Green Head  Gairdner  Hopetoun  Horrocks  Harrismith  Harvey  Hyden  Jerramungup  Jurien Bay  Kambalda  Katanning  Kalbarri  Kendenup  Kellerberrin  Kalannie  Kununoppin  Kojonup  Kondinin  Koorda  Kukerin  Kulin  Laverton  Ledge Point  Leeman  Leinster  Leonora  Lake Grace  Lake King  Lancelin  Latham  Meekatharra  Menzies  Munglinup  Miling  Mingenew  Marvel Loch  Manjimup  Moora  Merredin  Morangup  Margaret River  Mount Barker  Mt Magnet  Mukinbudin  Mullewa  Morawa  Nannup  Narrogin | Northcliffe  Newman  Northampton  Narembeen  Nungarin  Newdegate  Nyabing  Ocean Farm  Ongerup  Onslow  Paraburdoo  Pemberton  Perenjori  Pingrup  Pingelly  Port Gregory  Quairading  Ravensthorpe  Rocky Gully  Roebourne  Salmon Gums  Shark Bay  Southern Cross  Sandstone  Tambellup  Three Springs  Tammin  Tom Price  Toodyay  Trayning  Varley  Victoria Plains  Wagin  Walpole  Wellstead  Westonia  Wickham  Williams  Wongan Hills  Woodridge  Wickepin  Waroona  Wundowie  Wyalkatchem  Wyndham  Yalgoo  Yealering  York |

*Subject to operational requirements.
